# Targeting Oxidative Stress and Mitochondrial Dysfunction in Diabetic Neuropathy: Mechanisms and Therapeutic Opportunities

**DOI:** 10.3390/antiox15030367

**Published:** 2026-03-13

**Authors:** Ferenc Sztanek, László Imre Tóth, Marcell Hernyák, Attila Pető, Hajnalka Lőrincz, Adrienn Menyhárt, Dóra Marietta Balogh, Attila Csaba Nagy, Peter Kempler, György Paragh, Mariann Harangi

**Affiliations:** 1 Division of Metabolism, Department of Internal Medicine, Faculty of Medicine, University of Debrecen, H-4032 Debrecen, Hungary; toth.laszlo@med.unideb.hu (L.I.T.); hernyakmarcell@gmail.com (M.H.); paragh@belklinika.com (G.P.); harangi@belklinika.com (M.H.); 2 Doctoral School of Health Sciences, University of Debrecen, H-4032 Debrecen, Hungary; petoattilaa@gmail.com; 3 Third Department of Internal Medicine, Semmelweis Hospital of Borsod-Abauj-Zemplen County Central Hospital and University Teaching Hospital, H-3515 Miskolc, Hungary; 4 Department of Internal Medicine and Oncology, Faculty of Medicine, Semmelweis University, H-1085 Budapest, Hungary; menyhart.adrienn@stud.semmelweis.hu (A.M.); balogh.dora-marietta@semmelweis.hu (D.M.B.); kempler.peter@semmelweis.hu (P.K.); 5 Department of Health Informatics, Faculty of Health Sciences, University of Debrecen, H-4028 Debrecen, Hungary; attilanagy@med.unideb.hu; 6 Institute of Health Studies, Faculty of Health Sciences, University of Debrecen, H-4032 Debrecen, Hungary; 7 HUN-REN-UD Vascular Pathophysiology Research Group, University of Debrecen, H-4032 Debrecen, Hungary

**Keywords:** diabetic neuropathy, oxidative stress, mitochondrial dysfunction, inflammation, endothelial dysfunction, SGLT2 inhibitors, GLP-1 receptor agonists

## Abstract

Diabetic neuropathy is a frequent and disabling complication of diabetes, encompassing distal symmetric polyneuropathy and cardiovascular autonomic neuropathy, both associated with reduced quality of life and increased cardiovascular risk. Beyond its traditional interpretation as a direct consequence of chronic hyperglycaemia, oxidative stress has emerged as a central integrative mechanism linking metabolic overload, inflammation, mitochondrial dysfunction, and microvascular injury to progressive neural damage. These processes converge within the neurovascular unit, promoting a self-perpetuating cycle of axonal degeneration, impaired nerve perfusion and altered neuronal excitability. This narrative review synthesises experimental and clinical evidence on oxidative stress-related pathways implicated in diabetic neuropathy, including hyperglycaemia-activated metabolic routes, mitochondrial dysfunction, endoplasmic reticulum stress, and chronic inflammatory signalling. Classical antioxidant and mitochondrial-supportive interventions are evaluated alongside pleiotropic glucose-lowering agents, with particular emphasis on sodium–glucose cotransporter-2 inhibitors and glucagon-like peptide-1 receptor agonists, integrating mechanistic insights with biomarker and clinical outcome data. Conventional antioxidant strategies, such as α-lipoic acid, acetyl-L-carnitine, coenzyme Q10 and N-acetylcysteine, show reproducible benefits on neuropathic symptoms and oxidative stress markers, but evidence for sustained structural or disease-modifying effects remains limited. In contrast, incretin-based therapies and sodium–glucose cotransporter-2 inhibitors exert broader pleiotropic actions by attenuating oxidative and inflammatory signalling, improving mitochondrial homeostasis and endothelial function, with emerging evidence for modest but consistent neurophysiological and autonomic benefits. Overall, oxidative stress emerges as a key mechanistic hub in diabetic neuropathy. Future progress will depend on mechanism-aligned, neuropathy-specific clinical trials incorporating multidimensional endpoints and validated biomarkers.

## 1. Introduction

Diabetic neuropathy is the most common and earliest microvascular complication of diabetes, affecting more than 50% of patients. The clinical presentation of diabetic neuropathy is heterogeneous, with the most common forms being distal sensorimotor polyneuropathy (DSPN), cardiovascular autonomic neuropathy and diabetic mononeuropathies [1]. DSPN can have significant clinical consequences due to sensory loss, including an increased risk of foot ulcers and subsequent amputation in 15–40% of affected patients. Autonomic involvement further amplifies cardiovascular risk, contributing to a two- to three-fold increase in mortality. Despite the significant impact of diabetic neuropathy on quality of life and cardiovascular outcomes, it is frequently underdiagnosed and inadequately treated in diabetes [2].

Chronic hyperglycaemia, dyslipidaemia and insulin resistance activate a network of converging metabolic pathways implicated in the pathogenesis of diabetic neuropathy, including increased polyol pathway flux, protein kinase C activation, increased formation of advanced glycation end products (AGEs), and activation of the hexosamine pathway [3,4]. These processes converge on excessive mitochondrial production of reactive oxygen species (ROS), leading to oxidative stress, impaired axonal transport, neuroinflammation, and endothelial dysfunction in the vasa nervorum. Accumulating evidence indicates that oxidative stress is not merely a downstream consequence of metabolic disturbance but constitutes a central, self-perpetuating pathogenic mechanism linking metabolic injury to irreversible neural damage [5].

**Figure 1:**
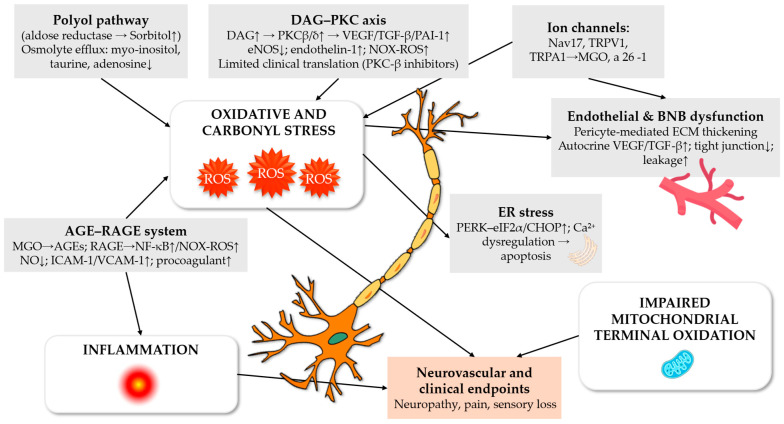
**Oxidative stress-centred pathogenetic and therapeutic framework of distal symmetric polyneuropathy (DSPN).** The figure summarises key oxidative stress-driven mechanisms underlying DSPN, highlighting mitochondrial dysfunction as a central downstream pathway of chronic hyperglycaemia. Excess mitochondrial reactive oxygen species contribute to endoneurial hypoxia, Schwann-cell dysregulation, endoplasmic reticulum stress, impaired myelination and progressive axonal damage. The therapeutic map outlines adjunctive redox- and mitochondria-targeted agents, targeted pathway inhibitors and “beyond-glycaemia” pharmacotherapies. Antioxidant and mitochondrial-supportive compounds are associated with relatively consistent symptomatic improvement, while structural or electrophysiological recovery remains variable. Incretin-based therapies and sodium–glucose cotransporter-2 inhibitors may exert indirect neuroprotective effects through systemic metabolic optimisation, reduction in oxidative stress and improvement of mitochondrial function. Overall, the framework emphasises the multifactorial nature of DSPN and the dissociation between symptomatic benefit and durable nerve regeneration. Abbreviations: DSPN, distal symmetric polyneuropathy; ROS, reactive oxygen species.

In parallel, chronic low-grade inflammation has emerged as a critical amplifier of redox-mediated neural injury. Pro-inflammatory cytokines, including tumour necrosis factor-α (TNF-α), interleukin-1β (IL-1β), and interleukin-6 (IL-6), appear to converge on inflammasome-dependent signalling pathways, with activation of the NOD-like receptor protein-3 (NLRP3) inflammasome representing a central regulatory node that amplifies nuclear factor-κB (NF-κB) activity, promotes sustained oxidative stress, impairs endothelial function and facilitates apoptosis within vulnerable neuronal populations [6]. Concurrent inactivation of nitric oxide by superoxide and peroxynitrite formation further compromises microvascular perfusion, exacerbating nerve ischaemia and demyelination [7]. Collectively, these mechanisms establish a tightly coupled redox–inflammatory–vascular axis that drives the progressive and largely irreversible nature of diabetic neuropathy (Figure 1).

From a therapeutic perspective, current management strategies for diabetic neuropathy remain predominantly symptomatic. Intensive glycaemic control delays the onset of neuropathy in type 1 diabetes (T1D). Nevertheless, its effectiveness in type 2 diabetes (T2D) is moderate and its benefits in advanced diabetic neuropathy are limited [8]. Symptomatic pharmacological treatments such as anticonvulsants, antidepressants and topical agents alleviate neuropathic pain but do not influence the underlying disease trajectory. Pathogenetically oriented therapies, including α-lipoic acid (ALA), the thiamine derivative benfotiamine and acetyl-L-carnitine (ALCAR), exert favourable antioxidant and metabolic effects and have consistently demonstrated short-term symptomatic benefits [9]. While definitive evidence for sustained structural disease modification is still limited, these pleiotropic actions support a biologically plausible potential to beneficially influence the course of diabetic neuropathy.

The management of painful DSPN typically involves pharmacotherapy with agents such as pregabalin, gabapentin, duloxetine, or topical capsaicin. In cases of severe, treatment-refractory pain, the use of opioid analgesics may be considered. In autonomic neuropathy, strict glycaemic control remains fundamental and may be complemented by pathogenetic interventions. However, the long-term disease-modifying efficacy of these strategies remains uncertain, underscoring the persistent therapeutic gap and the urgent need for interventions targeting the underlying pathogenic mechanisms rather than simply suppressing symptoms [9].

In this context, increasing attention has turned toward antidiabetic agents with pleiotropic actions that extend beyond glucose lowering. Sodium–glucose cotransporter-2 inhibitors (SGLT2i) and glucagon-like peptide-1 receptor agonists (GLP-1 RA) have been shown to attenuate oxidative stress, suppress inflammatory signalling, improve mitochondrial homeostasis and restore endothelial function in experimental and clinical settings. These properties position such agents as candidates capable of targeting the integrated pathogenic networks underlying diabetic neuropathy, rather than isolated downstream pathways [9].

Accordingly, the objective of this review is to synthesise current mechanistic and clinical evidence implicating oxidative stress and inflammation in the pathogenesis of diabetic neuropathy and to critically evaluate both classical antioxidant interventions and emerging pleiotropic antidiabetic agents. By integrating molecular mechanisms with clinical outcomes and biomarker data, our objective is to identify realistic pathways towards disease-modifying intervention in diabetic neuropathy, thereby establishing priorities for future neuropathy-focused clinical trials. This narrative review integrates mechanistic, biomarker and clinical evidence to evaluate oxidative stress-targeted strategies with a focus on disease modification in diabetic neuropathy.

### Literature Search Strategy

This article was designed as a narrative review integrating mechanistic, translational, and clinical evidence related to oxidative stress and mitochondrial dysfunction in diabetic neuropathy. The literature search was performed using PubMed/MEDLINE, Scopus and Web of Science databases. The primary focus was placed on publications from approximately the past 10–15 years, although earlier landmark studies were included when necessary to contextualise key mechanistic concepts. Priority was given to experimental studies elucidating pathophysiological mechanisms, human clinical trials, meta-analyses and high-quality reviews addressing oxidative stress pathways, mitochondrial dysfunction and therapeutic strategies relevant to diabetic neuropathy. The final selection of references was guided by relevance to the mechanistic framework and translational implications discussed in this review (Supplementary Text S1).

## 2. Pathophysiological Framework of Diabetic Neuropathy

### 2.1. Metabolic Overload and Redox Imbalance

As a multifactorial disorder, diabetic neuropathy arises from the convergence of metabolic, inflammatory, and vascular abnormalities that collectively result in oxidative stress and mitochondrial dysfunction within peripheral nerves. Chronic hyperglycemia diverts excess intracellular glucose into alternative metabolic pathways, including the polyol, diacylglycerol–protein kinase C (DAG-PKC), hexosamine, and receptor for advanced glycation end products axes [10]. Concomitant dyslipidemia and insulin resistance further exacerbate redox imbalance and impair endothelial integrity, thereby reinforcing neurovascular dysfunction in peripheral nerves [11]. These pathways do not act in isolation, but rather converge through shared downstream effectors, including mitochondrial ROS generation, inflammatory signalling and microvascular dysfunction. Therefore, it reinforces a self-perpetuating vicious cycle of neuronal damage (Table 1).

### 2.2. Polyol Pathway and NADPH Depletion

Within the polyol pathway, excess glucose is reduced to sorbitol by aldose reductase, a reaction that consumes nicotinamide adenine dinucleotide phosphate (NADPH) and consequently impairs the regeneration of the reduced glutathione. Intracellular accumulation of sorbitol induces osmotic stress and promotes the efflux of key osmolytes, including myo-inositol and taurine, thereby disrupting Na^+^/K^+^-ATPase activity and impairing axonal conduction [12]. In parallel, increased polyol flux enhances mitochondrial superoxide generation and accelerates depletion of endogenous antioxidant defences [7,12].

Experimental studies demonstrate that aldose reductase inhibition restores glutathione homeostasis through activation of nuclear factor erythroid 2-related factor 2 (Nrf2)-dependent transcriptional programmes in Schwann and endothelial cells [13]. Although the polyol pathway is considered to be a source of oxidative stress and has mechanistically received significant amounts of research [7,12], clinical experience indicates that the inhibition of a single pathway alone is not sufficient to reverse established neuropathy. This highlights the necessity for interventions that simultaneously address mitochondrial and vascular consequences.

### 2.3. DAG–PKC Signalling and Endothelial Dysfunction

Excessive glucose accumulation results in sustained activation of PKC isoforms, particularly PKC-β, disrupting neuronal and endothelial homeostasis [14,15]. PKC activation upregulates the expression of plasminogen activator inhibitor-1 (PAI-1), vascular endothelial growth factor (VEGF) and transforming growth factor-β (TGF-β), while suppressing Na^+^/K^+^-ATPase activity and endothelial nitric oxide synthase (eNOS) expression [15,16]. Concurrently, endothelin-1 signalling and activation of NADPH oxidase enhance superoxide production, thereby exacerbating vasoconstriction in the vasa nervorum [15,17]. Although PKC-β inhibition has been demonstrated in experimental models to reduce NF-κB activation and oxidative stress, clinical trials have failed to demonstrate sustained neuropathic benefit, underscoring the necessity for multifactorial therapeutic strategies [14,17].

### 2.4. Hexosamine Pathway and Aberrant O-GlcNAcylation

Excess glucose entry into the hexosamine biosynthesis pathway via glutamine fructose-6-phosphate amidotransferase (GFAT) results in increased production of UDP-N-acetylglucosamine and enhanced O-GlcNAcylation [18]. In hyperglycaemic states, O-GlcNAcylation of transcription factors such as specificity protein-1 promotes expression of pro-fibrotic and pro-inflammatory genes, including PAI-1 and TGF-β [19]. Additionally, aberrant O-GlcNAcylation impairs insulin signalling and eNOS signalling, thereby establishing a connection between hexosamine pathway flux and endothelial dysfunction. Preclinical and cellular studies indicate that the suppression of GFAT or other hexosamine pathway enzymes leads to a decrease in O-GlcNAc levels and a reduction in deleterious signalling [18]. Although hexosamine pathway activation contributes to vascular and inflammatory injury, its pathogenic relevance is largely mediated through convergence on oxidative and endothelial stress, reinforcing the concept of redox imbalance as a final common pathway.

### 2.5. AGE–RAGE Axis and Carbonyl Stress

Non-enzymatic glycation and accumulation of reactive dicarbonyls such as methylglyoxal lead to the formation of AGEs, which engages the receptor for AGEs (RAGE) on neurons, Schwann cells and endothelial cells [20]. The activation of RAGE triggers NF-κB signalling, NADPH oxidase-dependent ROS generation and amplification of inflammatory cytokine release. In addition, RAGE engagement involves the Ras and PKC pathways, thereby amplifying oxidative stress, cytokine release and apoptosis [20,21]. AGEs further impair nitric oxide bioavailability, increase endothelial adhesion molecule expression, and promote microvascular dysfunction within the endoneurium [22]. Clinical studies demonstrate correlations between AGE accumulation and the severity of diabetic neuropathy, underscoring translational relevance [23]. The AGE–RAGE axis acts as a potent amplifier of oxidative and inflammatory signalling, yet therapeutic modulation appears most effective when downstream redox and vascular injury are simultaneously addressed.

### 2.6. Chronic Inflammation as a Redox Amplifier

In the peripheral nerves, an imbalance in redox signalling further amplifies chronic inflammation. Chemokines such as monocyte chemoattractant protein-1 and C-X-C motif chemokine ligand 10 recruit monocytes and macrophages and engage microglia, thereby aggravating oxidative stress, demyelination and nociceptor sensitisation. Conversely, counter-regulatory cytokine pathways such as IL-10 are downregulated, while TGF-β exhibits context-dependent modulation, collectively delaying inflammatory resolution [24,25]. Toll-like receptor signalling (particularly via TLR4) and activation of the NLRP3 inflammasome provide an upstream mechanistic link between metabolic stressors, including hyperglycaemia and dyslipidaemia and NF-κB activation in peripheral nerves. These pathways have been observed in Schwann cells and dorsal root ganglion neurons, where they couple glucotoxic and lipotoxic stress to sustained neuroinflammatory responses [24]. In parallel, a six-week aerobic exercise intervention in patients with T2D and DSPN increased circulating fibroblast growth factor 21 levels and was associated with improvements in current perception thresholds and inflammatory indices, supporting a mechanistic role for structured physical activity as a disease-modifying adjunct [26]. Inflammation in diabetic neuropathy is not an independent driver but an amplifier of redox-mediated injury, suggesting that isolated anti-inflammatory strategies are unlikely to succeed without concomitant correction of oxidative stress.

### 2.7. Mitochondrial Dysfunction and NAD^+^–PARP Axis

Mitochondria represent both a major source and target of ROS in diabetic neuropathy. In experimental models of painful DSPN, oxidative DNA damage induces overactivation of poly(ADP-ribose) polymerase-1 (PARP-1), leading to depletion of nicotinamide adenine dinucleotide (NAD^+^) and suppression of the sirtuin-1 and peroxisome proliferator-activated receptor γ coactivator-1α (PGC-1α) axis, thereby impairing mitochondrial biogenesis and mitophagy [27,28]. A subsequent ultrastructural analysis of human sural nerve biopsies from patients with diabetic sensory neuropathy has revealed swollen and structurally damaged mitochondria with fragmented cristae, consistent with a self-perpetuating cycle of energy deprivation and ROS leakage [29]. Further evidence from rodent models of diabetes demonstrates impaired mitochondrial respiration and reduced activity of electron transport chain complexes, particularly complexes I and IV, in dorsal root ganglion neurons. This results in reduced ATP synthesis, axonal mitochondrial depolarization and loss of transport. Notably, these deficits are largely reversed by insulin replacement [30,31]. Mitochondrial dysfunction represents a final common denominator across metabolic and inflammatory insults, providing a strong rationale for therapies capable of restoring mitochondrial homeostasis rather than targeting upstream pathways in isolation.

The neurovascular unit, including the vasa nervorum, is particularly susceptible to redox stress in diabetes. Excessive production of ROS promotes the rapid reaction of superoxide with NO, forming peroxynitrite and leading to eNOS uncoupling and reduced NO bioavailability. This process establishes a vicious cycle of oxidative and nitrative stress, further impairing endoneurial perfusion and axonal energy supply. AGEs exert additional deleterious effects on pericytes and endothelial cells through RAGE/NF-κB signalling, inducing basement membrane thickening and pericyte-mediated remodelling of the extracellular matrix. Moreover, AGEs compromise tight junction integrity and disrupt the blood–nerve barrier via autocrine VEGF and TGF-β signalling in pericytes, thereby facilitating inflammatory cell infiltration and further oxidative injury [32].

Research on NAD^+^ metabolism has identified PARP overactivation as a pivotal contributor to DSPN. Oxidative DNA damage induces excessive PARP activation, resulting in pathological NAD^+^ depletion and impairment of glycolysis, mitochondrial respiration and cellular stress responses [27,28]. In experimental models of diabetes, pharmacological or genetic inhibition of PARP attenuates intraepidermal nerve fibre loss and alleviates painful neuropathy [33]. PARP hyperactivation further diverts glycolytic intermediates toward collateral pathways under metabolic stress, thereby amplifying oxidative injury and reinforcing redox imbalance [34]. ROS-mediated vascular injury and PARP overactivation result in closely linked redox-energetic impairment at the level of the neurovascular unit. Consequently, therapeutic strategies that may preserve NAD^+^ homeostasis and endothelial function may offer benefits that extend beyond the scope of interventions targeting oxidative stress alone.

### 2.8. Endoplasmic Reticulum Stress and Unfolded Protein Response

Chronic hyperglycaemia and lipid abnormalities impose a sustained proteostatic burden on the endoplasmic reticulum (ER), activating the unfolded protein response (UPR) through the protein kinase RNA-like ER kinase (PERK)–eukaryotic initiation factor 2α/CEBP homologous protein (CHOP), inositol-requiring enzyme 1α, and activating transcription factor 6 signalling [35]. In peripheral nerves of diabetic rodent models, prolonged UPR activation shifts from an adaptive to a proapoptotic programme, amplifying oxidative stress, calcium dysregulation, and mitochondrial dysfunction. Notably, genetic ablation of CHOP in diabetic mice is associated with attenuation of neuropathy severity and reduced markers of oxidative and nitrative damage, implicating maladaptive UPR signalling as a contributor to disease progression [35]. These findings position ER stress not as an isolated mechanism but as an amplifier of redox-mediated neuronal injury under sustained metabolic stress.

In experimental models of diabetic neuropathy, administration of chemical chaperones such as trimethylamine-N-oxide and 4-phenylbutyric acid reduces markers of ER stress, restores redox homeostasis, and improves peripheral nerve function [35]. In recent studies, treatment with compound Qiying granules in DSPN rats has been shown to suppress PERK/CHOP pathway activation and improve axonal integrity and myelin morphology [36]. ER stress emerges as a convergent response to chronic hyperglycaemia, inflammation, and dyslipidaemia, propagating oxidative and mitochondrial dysfunction. Targeting ER stress alone is unlikely to suffice; however, interventions that alleviate metabolic overload and restore redox balance may indirectly normalise maladaptive UPR signalling.

### 2.9. Ion Channel Remodelling and Nociceptor Hyperexcitability

Diabetic neuropathy is characterised by activity-dependent remodelling of nociceptor ion channels under sustained metabolic, inflammatory, and oxidative stress. A substantial body of experimental and clinical evidence supports disease-related plasticity of voltage-gated sodium (Nav) channels, which contributes to altered excitability and pain phenotypes in DSPN. In streptozotocin-induced diabetes, Nav1.7 expression is increased in dorsal root ganglion neurons through a TNF-α/NF-κB-dependent mechanism and pharmacological or genetic inhibition of Nav1.7 attenuates pain-related behaviours [37]. In a broader context, Nav channel dysfunction represents a consistent electrophysiological signature of DSPN rather than an isolated molecular abnormality [38].

Transient receptor potential channels have also emerged as critical mediators linking metabolic stress to nociceptor hyperexcitability. The expression and function of transient receptor potential vanilloid-1 is altered in diabetic models in a phenotype-dependent manner, reflecting heterogeneity in painful versus painless neuropathy [39]. In parallel, vascular-coupled sensitization of transient receptor potential ankyrin-1 induces cold sensitivity during the early stages of painful DSPN [40]. Reactive carbonyl stress provides a mechanistic bridge between dysregulated metabolism and altered excitability, as methylglyoxal directly activates and sensitises transient receptor potential ankyrin-1, thereby promoting nociceptor hyperexcitability and spontaneous activity [41,42].

Converging neurophysiological evidence indicates that pathological states, including streptozocin-induced DSPN, are accompanied by spontaneous ectopic discharges in C-nociceptors, consistent with lowered activation thresholds and abnormal impulse generation [43]. These channel- and circuit-level alterations reflect downstream consequences of sustained redox, inflammatory, and metabolic stress rather than primary disease drivers. From a clinical perspective, this mechanistic understanding aligns with the efficacy of symptomatic therapies targeting neuronal excitability. Pregabalin has demonstrated an analgesic benefit in painful DSPN in randomised, placebo-controlled trials [44], while high-concentration topical capsaicin (8%) has demonstrated benefits across a range of chronic neuropathic pain conditions in controlled trials [45]. Importantly, these interventions modulate neuronal firing without altering the underlying metabolic or oxidative milieu that drives disease progression. While channel-targeted therapies effectively alleviate neuropathic pain, they do not address the upstream pathogenic networks responsible for neural degeneration, underscoring the need for disease-modifying strategies that restore metabolic, oxidative and vascular homeostasis.

To summarise, these mechanistic pathways converge on oxidative stress as a central integrative driver of diabetic neuropathy. While individual metabolic routes contribute to disease initiation and progression, their pathogenic impact is mediated through shared downstream processes, including mitochondrial dysfunction, chronic inflammation and microvascular injury. This convergence provides a robust biological rationale for pleiotropic therapeutic strategies capable of simultaneously modulating redox balance, inflammation and endothelial function.

### 2.10. Integrative Summary: Oxidative Stress as a Central Mechanism

The presence of oxidative stress and mitochondrial dysfunction has been identified as a pivotal contributing factor in the development of DSPN. These processes disrupt endoneurial microcirculation, Schwann-cell homeostasis, myelination and axonal transport [3,11]. Persistent hyperglycaemia promotes excessive mitochondrial ROS production and impairs oxidative phosphorylation in sensory neurons, thereby initiating a feedforward cycle of redox imbalance and energetic deficit [30,31]. Within this mechanistic framework, antioxidant and mitochondrial-targeted interventions represent biologically plausible strategies for symptom relief and partial pathway modulation. However, definitive evidence for durable structural or disease-modifying effects remains limited.

**Table 1:** The pathophysiological framework of diabetic neuropathy highlights oxidative stress as a central integrative mechanism.

Pathophysiological Domain	Key Molecular Pathways	Principal Oxidative/ Inflammatory Mediators	Neurovascular Consequences	Translational Relevance	References
Metabolic overload	Chronic hyperglycaemia, dyslipidaemia, insulin resistance	Mitochondrial ROS, carbonyl stress	Axonal energy deficit, impaired axonal transport	Rationale for early metabolic and pleiotropic intervention	[3,4,5,11]
Polyol pathway activation	Aldose reductase-mediated glucose reduction	NADPH depletion, reduced glutathione, superoxide	Osmotic stress, Na^+^/K^+^-ATPase dysfunction, slowed nerve conduction	Limited efficacy of single-pathway inhibition; supports combination strategies	[7,12,13]
DAG–PKC signalling	PKC-β activation, NADPH oxidase	Superoxide, reduced NO bioavailability	Endoneurial vasoconstriction, impaired microcirculation	Explains modest clinical impact of PKC-β inhibitors	[14,15,16,17]
Hexosamine pathway flux	GFAT activation, O-GlcNAcylation	Pro-inflammatory and pro-fibrotic gene expression	Endothelial dysfunction, impaired insulin signalling	Upstream contributor converging on oxidative stress	[18,19]
AGE–RAGE axis	Non-enzymatic glycation, RAGE signalling	NF-κB activation, ROS, cytokine amplification	Microvascular injury, blood–nerve barrier disruption	Biomarker-supported target, but insufficient alone	[20,21,22,23]
Chronic inflammation	NF-κB, NLRP3 inflammasome, cytokine cascades	TNF-α, IL-1β, IL-6, chemokines	Demyelination, nociceptor sensitisation	Acts as amplifier rather than primary driver	[6,24,25]
Mitochondrial dysfunction	Impaired ETC activity, reduced biogenesis	Excess ROS, reduced ATP	Axonal degeneration, impaired regeneration	Central therapeutic target across disease stages	[27,28,29,30,31]
PARP overactivation and NAD^+^ depletion	DNA damage-induced PARP activation	NAD^+^ loss, energetic failure	Axonal loss, small-fibre degeneration	Links oxidative stress to bioenergetic collapse	[27,28,33,34]
Endoplasmic reticulum stress	PERK–eIF2α–CHOP, IRE1α, ATF6	Proteostatic stress, secondary ROS	Schwann-cell dysfunction, apoptosis	Amplifies redox and mitochondrial injury	[35,36]
Ion channel remodelling	Nav, TRP channel plasticity	Carbonyl stress, inflammatory mediators	Hyperexcitability, neuropathic pain	Explains symptomatic efficacy of channel-targeted drugs	[37,38,39,40,41,42,43]
Neurovascular unit dysfunction	Endothelial–pericyte–axon uncoupling	Reduced NO, oxidative/nitrative stress	Endoneurial hypoxia, ischemia	Integrates vascular and neural injury	[7,22,32]

This table summarises the principal pathophysiological domains contributing to diabetic neuropathy, with emphasis on oxidative stress as a shared downstream mediator within the neurovascular unit. Metabolic, inflammatory, mitochondrial, and vascular pathways converge on redox imbalance, mitochondrial dysfunction, and microvascular impairment, thereby driving axonal injury, demyelination, and altered neuronal excitability. The framework provides a biological rationale for pleiotropic and combination-based therapeutic strategies that extend beyond single-pathway inhibition. Abbreviations: AGE, advanced glycation end products; ATF6, activating transcription factor 6; ATP, adenosine triphosphate; CHOP, C/EBP homologous protein; DAG, diacylglycerol; eIF2α, eukaryotic initiation factor 2 alpha; ETC, electron transport chain; GFAT, glutamine–fructose-6-phosphate amidotransferase; IL-1β, interleukin-1 beta; IL-6, interleukin-6; IRE1α, inositol-requiring enzyme 1 alpha; Nav, voltage-gated sodium channels; NAD^+^, nicotinamide adenine dinucleotide; NADPH, nicotinamide adenine dinucleotide phosphate; NF-κB, nuclear factor kappa B; NLRP3, NOD-like receptor family pyrin domain-containing 3; NO, nitric oxide; O-GlcNAcylation, O-linked β-N-acetylglucosamine modification; PARP, poly(ADP-ribose) polymerase; PERK, protein kinase RNA-like endoplasmic reticulum kinase; PKC, protein kinase C; PKC-β, protein kinase C beta isoform; RAGE, receptor for advanced glycation end products; ROS, reactive oxygen species; TNF-α, tumour necrosis factor alpha; TRP, transient receptor potential channels.

## 3. Therapeutic Strategies Targeting Oxidative Stress

### 3.1. Classical Antioxidant and Mitochondrial-Supportive Therapies

#### 3.1.1. Alpha-Lipoic Acid

ALA, a dithiol antioxidant and mitochondrial cofactor, has demonstrated short-term symptomatic benefits in randomised controlled trials and meta-analyses, particularly when administered intravenously at a dose of 600 mg/day for approximately three weeks. Nevertheless, effects on nerve conduction velocity and long-term outcomes remain variable [46,47,48]. The efficacy of oral ALA supplementation, administered at daily doses ranging from 600 to 800 mg, exhibits heterogeneous efficacy across studies, although its overall safety profile is favourable [49,50]. In the ORPIL (oral pilot) study, patients with DSPN receiving oral ALA at a dose of 600 mg three times daily for three weeks demonstrated a significant reduction in neuropathic symptom burden and an improvement in neurological deficit scores compared with placebo, supporting a biologically relevant modulation of oxidative stress-related pathways beyond purely symptomatic analgesic effects [51]. Preclinical data align with these observations, demonstrating increased endoneurial perfusion, partial correction of sensory and motor nerve conduction deficits, and attenuation of oxidative injury in diabetic rodent models [52,53,54,55].

**Figure 2:**
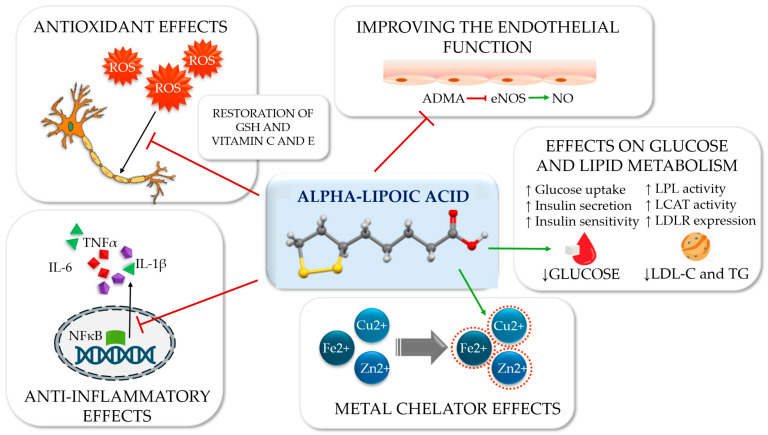
**Multimodal biological effects of α-lipoic acid relevant to diabetic neuropathy.** This figure illustrates the pleiotropic biological actions of α-lipoic acid relevant to the pathophysiology of diabetic neuropathy. Antioxidant effects include direct scavenging of reactive oxygen species and regeneration of endogenous antioxidant systems, such as glutathione and vitamins C and E. α-Lipoic acid further improves endothelial function by modulating the ADMA–eNOS–nitric oxide axis, thereby supporting microvascular perfusion. Additional mechanisms include anti-inflammatory effects mediated through inhibition of NF-κB signalling and downstream pro-inflammatory cytokine production, as well as favourable effects on glucose and lipid metabolism, contributing to improved insulin sensitivity and lipid handling. Metal chelation of redox-active transition metals represents an additional pathway by which oxidative stress amplification may be attenuated. Collectively, these mechanisms support the role of α-lipoic acid as an adjunctive, mechanism-oriented intervention rather than a primary disease-modifying therapy. Abbreviations: ADMA, asymmetric dimethylarginine; eNOS, endothelial nitric oxide synthase; NO, nitric oxide; NF-κB, nuclear factor kappa B; TNF-α, tumour necrosis factor alpha; IL-1β, interleukin-1 beta; IL-6, interleukin-6; LPL, lipoprotein lipase; LCAT, lecithin–cholesterol acyltransferase; LDLR, low-density lipoprotein receptor; LDL-C, low-density lipoprotein cholesterol; TG, triglycerides.

Biomarker-based studies further support the biological plausibility of ALA therapy. Progranulin is a pleiotropic mediator implicated in inflammatory modulation and neuronal repair processes, and its circulating concentrations increased after six months of daily treatment with 600 mg of α-lipoic acid, accompanied by an improvement in current perception threshold in patients with DSPN [56]. Concurrent reductions in asymmetric dimethylarginine (ADMA), an endogenous inhibitor of eNOS, have been reported in diabetic cohorts receiving ALA [57,58]. A subsequent cohort study also demonstrated reduced AGE accumulation, correlating with sensory improvement and attenuation of inflammatory and endothelial markers [59]. In the NATHAN 1 trial, long-term administration of oral ALA (600 mg/day) over four years in patients with mild to moderate DSPN did not significantly improve the primary composite endpoint; however, clinically meaningful improvements and a slowing of neurological progression were observed across several secondary neuropathy measures, consistent with a stage-dependent influence on underlying pathogenetic mechanisms and favourable tolerability. These findings support the hypothesis that ALA represents a biologically coherent adjunctive therapy with reproducible symptomatic benefits and favourable vascular–redox effects [49,60] (Figure 2).

#### 3.1.2. Acetyl-L-Carnitine

ALCAR has been demonstrated to exert therapeutic effects in the treatment of DSPN through modulation of mitochondrial β-oxidation and enhancement of neurotrophic signalling. Experimental studies indicate that ALCAR upregulates nerve growth factor expression and improves neurotrophin receptor responsiveness, thereby supporting neuronal survival and regeneration in diabetic conditions [61,62]. A systematic review and meta-analysis of randomised controlled trials reported modest but statistically significant reductions in neuropathic pain with oral or intramuscular ALCAR administered at doses of 1–3 g/day [61]. In addition to its analgesic effects, a comprehensive synthesis of evidence in painful peripheral neuropathies demonstrated an approximate 20% reduction in visual analogue scale pain scores from baseline, alongside improvements in nerve conduction velocity and indices of small-fibre regeneration [62]. However, a Cochrane systematic review specifically focusing on DSPN rated the certainty of pain relief at 6–12 months as low or very low, reflecting the heterogeneity of study design and limited long-term data [63]. These analyses indicate that ALCAR demonstrates consistent neurotrophic and mitochondrial rationale with modest symptomatic efficacy. Nonetheless, current evidence is insufficient to support classification as a disease-modifying intervention in DSPN.

#### 3.1.3. Coenzyme Q10

Coenzyme Q10, a mitochondrial electron transport chain cofactor with antioxidant properties, has demonstrated a clinical benefit in painful DSPN. In a randomised, placebo-controlled trial, coenzyme Q10 supplementation at 300 mg/day for eight weeks, administered alongside pregabalin (150 mg/day), resulted in greater pain relief, improved sleep disturbance scores and a higher proportion of ≥50% pain responders compared with placebo [64]. Preclinical studies support these findings, showing prevention of nerve conduction impairment and reduction in dorsal root ganglion neuronal loss in db/db mice following early, chronic coenzyme Q10 administration [65]. Coenzyme Q10 may potentiate symptomatic treatment and confer neuroprotective effects when administered early; however, evidence for sustained structural benefit in established DSPN remains limited.

#### 3.1.4. N-Acetylcysteine

N-acetylcysteine (NAC), a glutathione precursor with antioxidant and anti-inflammatory properties, targets the convergence of oxidative stress and inflammation in diabetic neuropathy. In an eight-week, double-blind, randomised, placebo-controlled trial, the combination of NAC (1200 mg/day) and pregabalin (150 mg/day) therapy was found to significantly reduce neuropathic pain and sleep disturbance scores, as well as the ≥50% pain response rate, in comparison with the placebo. Furthermore, shifts in biomarkers were concomitant with increased antioxidant engagement as demonstrated by reductions in malondialdehyde and increases in superoxide dismutase, glutathione peroxidase, total antioxidant capacity and thiol levels [66]. Preclinical studies corroborate these findings, demonstrating preservation of motor nerve conduction velocity and attenuation of myelin pathology in streptozotocin-induced diabetic rats following oral NAC administration [67]. Collectively, these observations lend support to the notion that NAC should be considered a biologically acceptable adjunctive therapy until the results of larger and longer-term studies are available to ascertain its durability and disease-modifying potential.

#### 3.1.5. B Vitamins and Methylcobalamin

**Figure 3:**
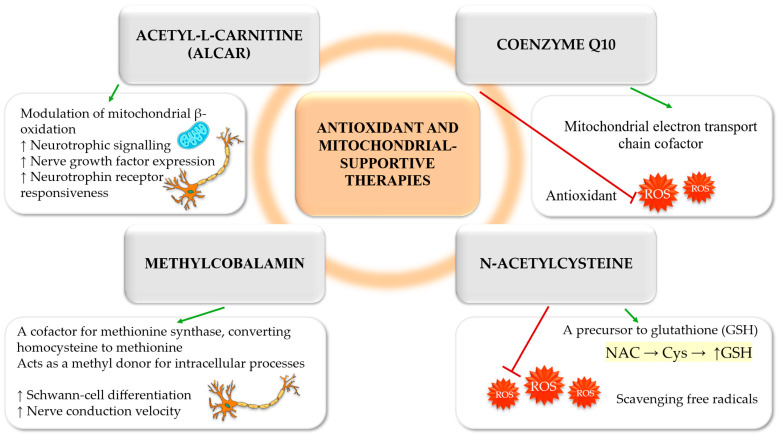
**Antioxidant and mitochondrial-supportive adjunctive therapies in diabetic neuropathy.** This figure summarises selected adjunctive therapies targeting mitochondrial dysfunction and oxidative stress in diabetic neuropathy. Acetyl-L-carnitine modulates mitochondrial β-oxidation and enhances neurotrophic signalling, supporting neuronal maintenance and nerve repair. Coenzyme Q10 functions as a cofactor of the mitochondrial electron transport chain and exerts antioxidant effects, thereby limiting reactive oxygen species generation. Methylcobalamin acts as a cofactor for methionine synthase and as a methyl donor in intracellular processes, promoting Schwann-cell differentiation and nerve conduction. N-acetylcysteine serves as a precursor for glutathione synthesis and directly scavenges free radicals. Collectively, these agents support mitochondrial homeostasis and redox balance, providing symptomatic benefits as adjuncts rather than definitive disease-modifying therapies. Abbreviations: ALCAR, acetyl-L-carnitine; NAC, N-acetylcysteine; ROS, reactive oxygen species; GSH, glutathione.

B vitamins, particularly methylcobalamin (vitamin B12), have been investigated as potential therapeutic agents for the treatment of DSPN. In a 24-week, multicentre, randomised, double-blind trial, oral methylcobalamin (1.5 mg/day) was non-inferior to ALCAR (1500 mg/day) in improving neuropathic symptoms, disability scores and nerve conduction velocity [68]. Meta-analyses further indicate that combination therapy with methylcobalamin and ALA provides greater symptomatic and neurophysiological benefit than methylcobalamin monotherapy over treatment durations of 2–4 weeks [8]. From a mechanistic perspective, ultra-high-dose methylcobalamin accelerates axonal regeneration in experimental neuropathy models, reinforcing biological plausibility [69]. Vitamin B12 supplementation is justified in deficient states and as part of multimodal regimens, yet evidence for independent disease modification in DSPN remains limited (Figure 3).

#### 3.1.6. Clinical Interpretation and Limitations

Collectively, classical antioxidant strategies, including ALA, ALCAR, coenzyme Q10, NAC, and high-dose methylcobalamin, have demonstrated reproducible benefits in neuropathic pain, sleep disturbance and patient-reported outcomes. In clinical practice, these agents are best positioned as adjuncts to standard DSPN management, encompassing optimisation of glycaemic control, lipid and blood pressure management, correction of B12 vitamin deficiencies, and evidence-based analgesic therapy [70]. Future priorities include adequately powered, long-duration randomised controlled trials focused specifically on DSPN, employing harmonised endpoint sets that integrate patient-centred outcomes with objective neurophysiological and small-fibre assessments.

### 3.2. Phytochemicals and Hormonal Antioxidants

**Figure 4:**
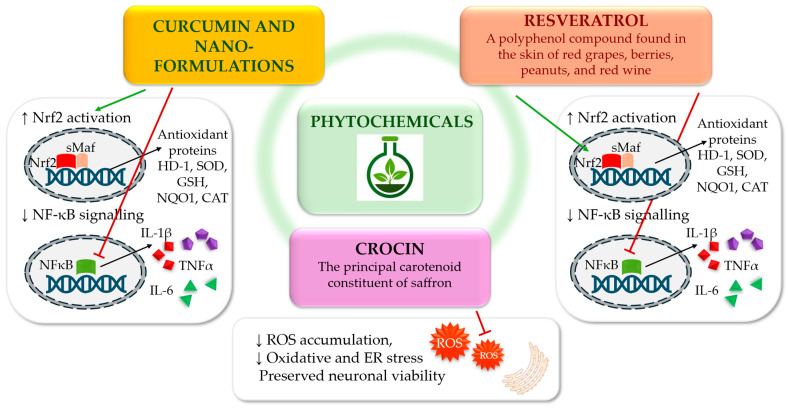
**Redox- and inflammation-modulating effects of selected phytochemicals relevant to diabetic neuropathy.** This figure summarises the shared and compound-specific mechanisms by which selected phytochemicals—curcumin (including nano-formulations), resveratrol, and crocin—modulate oxidative stress and inflammatory signalling pathways relevant to diabetic neuropathy. These agents primarily activate the Nrf2–sMaf transcriptional axis, leading to upregulation of endogenous antioxidant and cytoprotective proteins, while concurrently suppressing NF-κB-dependent pro-inflammatory signalling and cytokine production. Downstream effects include attenuation of reactive oxygen species accumulation, reduction in oxidative and endoplasmic reticulum stress, and preservation of neuronal viability. The schematic highlights phytochemicals as adjunctive, mechanism-oriented interventions with biological plausibility, while acknowledging limitations related to bioavailability, formulation, and translational consistency. Abbreviations: ROS, reactive oxygen species; Nrf2, nuclear factor erythroid 2-related factor 2; sMaf, small musculoaponeurotic fibrosarcoma proteins; NF-κB, nuclear factor kappa B; IL-1β, interleukin-1 beta; IL-6, interleukin-6; TNF-α, tumour necrosis factor alpha; SOD, superoxide dismutase; GSH, glutathione; NQO1, NAD(P)H quinone dehydrogenase 1; CAT, catalase; ER, endoplasmic reticulum.

Within the complex pathophysiology of diabetic neuropathy, phytochemicals and the hormonal antioxidant melatonin have been proposed as modulators of redox–inflammatory signalling and endothelial dysfunction at multiple levels. This concept aligns with the broader complementary literature on diabetic neuropathy where the majority of reported clinical benefits are adjuvant and symptomatic in nature rather than disease-modifying. Improvements in neuropathic pain, sleep quality, and patient-reported outcomes are the most consistently reported effects, whereas sustained neurophysiological or morphological benefits remain uncertain across studies [71] (Figure 4).

#### 3.2.1. Melatonin

Melatonin has been shown to possess direct free radical scavenging activity and to enhance endogenous antioxidant defences, in addition to antinociceptive effects mediated through its own receptor-related pathways. These properties have been consistently demonstrated in experimental models of neuropathic pain [71,72,73]. In a randomised, double-blind, placebo-controlled trial involving patients with painful polyneuropathy of diabetic and non-diabetic origin, adjunctive melatonin titrated from 3 mg to 6 mg daily in combination with pregabalin (150 mg/day) resulted in significant reductions in pain intensity and sleep disturbance scores, alongside higher responder rates compared with placebo, with good tolerability [74]. In contrast, a more recent crossover, randomised, controlled trial in mixed neuropathic pain populations failed to demonstrate superiority of melatonin over placebo [75]. It is hypothesised that melatonin exhibits consistent antinociceptive and sleep-modulating effects, yet the current evidence does not support disease-modifying efficacy in DSPN. This emphasises the necessity for indication-specific and neuropathy-focused study designs.

#### 3.2.2. Curcumin and Nano-Formulations

Curcumin has been demonstrated to target key redox and inflammatory signalling pathways, including the activation of Nrf2/Kelch-like ECH-associated protein 1 and the inhibition of NF-κB. These effects translate into antinociceptive and neuroprotective signals in multiple rodent models of neuropathy [76]. In an early randomised, double-blind clinical trial in patients with T2D and DSPN, nano-formulated curcumin at 80 mg/day for eight weeks reduced overall neuropathy severity and reflex scores compared to placebo, accompanied by modest improvements in glycaemic indices [77]. In contrast, a larger 16-week, double-blind, randomised, controlled trial using 40 mg nano-formulated curcumin twice daily did not demonstrate any improvement over placebo for pain scores, neuropathy disability indices, or Michigan Neuropathy Screening Instrument outcomes [78]. Despite mechanistic plausibility and early symptomatic signals, nano-formulated curcumin has not demonstrated consistent clinical benefits across adequately powered trials and evidence for disease modification in DSPN remains insufficient.

#### 3.2.3. Resveratrol

Resveratrol exhibits antioxidant, anti-inflammatory, and analgesic properties relevant to DSPN. In preclinical models of DSPN, resveratrol has been shown to reduce painful neuropathy and structural nerve damage while activating Nrf2 and suppressing NF-κB signalling pathways [79,80]. Additionally, resveratrol has been observed to reduce transient receptor potential channel-mediated calcium influx [80]. However, randomised clinical evidence specific to DSPN remains limited. In patients with T2D without neuropathy-specific endpoints, an eight-week randomised, double-blind trial demonstrated that resveratrol supplementation (800 mg/day) improved systemic and cellular oxidative stress markers [81]. Meta-analyses of randomised trials in T2D report modest improvements in glycaemic control and cardiometabolic parameters [82]. These results indicate that resveratrol displays both biological plausibility and robust mechanistic support. However, the clinical evidence in DSPN remains preliminary and requires validation through neuropathy-specific outcome trials.

#### 3.2.4. Crocin

Crocin, the principal carotenoid constituent of saffron (Crocus sativus), has recently been evaluated in a triple-blind, randomised, placebo-controlled trial in patients with T2D and DSPN. Over a 12-week treatment period, crocin administered at 15 mg twice daily significantly reduced neuropathic pain intensity and total symptom scores, with a favourable safety profile [83]. While these preliminary results suggest a possible therapeutic role for crocin in the treatment of diabetic neuropathy, further investigation is necessary to ascertain the durability of clinical response, dose–response characteristics and objective neurophysiological outcomes [84]. Mechanistic and in vitro studies support these clinical findings: in models of high glucose-induced neuronal damage, crocin reduced ROS accumulation, suppressed oxidative and ER stress and preserved neuronal viability, providing a biologically plausible antioxidant and neuroprotective mechanism [84,85]. While early clinical signals are promising, the durability of crocin’s effects and its impact on objective neurophysiological endpoints remain to be established in larger, longer-term trials.

#### 3.2.5. Integrative Perspective on Phytochemicals and Hormonal Antioxidants

Across phytochemical and hormonal antioxidants, including nano-curcumin, resveratrol, melatonin, and crocin, the most consistent results in human trials are adjuvant and symptomatic effects, including improvements in pain, sleep disturbances and patient-reported outcomes. Structural, neurophysiological, or disease-modifying effects remain inconsistent and insufficiently substantiated. Accordingly, these agents are best positioned as adjuncts to standard DSPN management, which prioritises optimisation of metabolic risk factors and evidence-based analgesic therapy [70]. Future research priorities include adequately powered, longer-term, randomised, DSPN-specific trials that integrate neuropathic pain assessment with objective measures of small-fibre structure and function as well as nerve conduction parameters. In parallel, mechanistic biomarker studies linking redox and cytokine modulation to clinical benefits will be essential to validate these interventions and facilitate translation towards credible disease-modifying strategies [74,86].

### 3.3. Incretin-Based Therapies with Antioxidant Effects

Incretin-based therapies have attracted increasing interest in diabetic neuropathy due to their pleiotropic actions at the intersection of metabolic regulation, oxidative stress, chronic inflammation and endothelial dysfunction, thereby offering a biologically plausible framework for disease-modifying intervention.

#### 3.3.1. Dipeptidyl Peptidase-4 Inhibitors

Dipeptidyl peptidase-4 inhibitors (DPP-4is) exhibit antifibrotic, anti-inflammatory, and antioxidant properties that extend beyond glucose lowering [87]. These mechanisms have been implicated in the potential for renal and vascular protection. At the endothelial level, DPP-4i has been shown to enhance endothelial function, increase NO bioavailability, and attenuate oxidative stress. These effects have been consistently demonstrated in preclinical models and have been confirmed to varying degrees in human studies [88].

Despite the absence of targeted randomised controlled trials in DSPN, a small, open-label pilot study has suggested the potential efficacy of teneligliptin in enhancing pseudomotor function and measures of autonomic and peripheral neuropathy. Nevertheless, the open-label design and relatively modest sample size of the study have been emphasised as notable methodological limitations [89]. The effects of DPP-4i on oxidative stress attenuation, cytokine modulation and endothelial dysfunction, as well as the mechanistic overlap between canonical pathways underlying endoneurial hypoxia and axonal loss, provide a biologically plausible rationale for evaluating DPP-4i as an adjunctive treatment in diabetic neuropathy. However, this rationale requires further validation through adequately powered, disease-specific randomised trials [87,88].

#### 3.3.2. Glucagon-like Peptide-1 Receptor Agonists

GLP-1 RAs have been demonstrated to activate convergent neuroprotective and vasculoprotective pathways highly relevant to DSPN pathogenesis. In streptozotocin-induced diabetic rats, liraglutide suppressed p38 mitogen-activated protein kinase and NF-κB signalling in the sciatic nerve, accompanied by reductions in TNF-α, IL-1β, and IL-6 expression independent of glycaemic control [90]. In human studies, GLP-1 administration mitigated hyper- and hypoglycaemia-induced elevations in circulating matrix metalloproteinase-9 (MMP-9), consistent with antioxidant and endothelial-protective effects [91].

Beyond cytokine modulation, GLP-1 RA therapy improved leukocyte redox status, mitochondrial function and endothelial–leukocyte interactions in T2D, supporting restoration of microvascular homeostasis [92,93]. These findings indicate a correlation between GLP-1 RA-mediated attenuation of NF-κB-driven cytokine signalling and oxidative stress with downstream suppression of MMP-9 activity and preservation of microvascular homeostasis. It is hypothesised that these mechanisms enhance nerve perfusion and neuronal survival [90,91,93].

Preliminary clinical data is largely consistent with the underlying mechanistic rationale. A meta-analysis reported modest improvements in nerve conduction velocity with GLP-1 RA therapy, apparently independent of glycaemic changes [94]. Structural outcomes show similar signals: in a prospective observational study, initiation of semaglutide or dulaglutide was associated with partial normalisation of peripheral nerve morphology on ultrasound and with increases in sural sensory amplitudes [95]. Additional exploratory evidence from corneal confocal microscopy demonstrated increases in corneal nerve fibre density and length following semaglutide treatment in children with obesity due to melanocortin-4 receptor mutations; however, confirmation in DSPN-specific cohorts is lacking [96].

Conversely, a 26-week randomised, double-blind trial in adults with T1D and established neuropathy showed that liraglutide reduced IL-6 concentrations without improving autonomic or peripheral neurophysiological outcomes, highlighting heterogeneity across disease phenotypes and trial designs [97]. A synthesis of evidence on neurological complications similarly concludes that data on DSPN remain limited and inconsistent, particularly regarding pain and autonomic endpoints [98].

Meta-analyses and mechanistic studies consistently report modest increases in resting heart rate with GLP-1 RAs, an effect warranting caution in patients with cardiovascular autonomic neuropathy or advanced cardiac disease [99,100]. These observations underscore the importance of phenotype-specific patient selection in future DSPN trials.

#### 3.3.3. Integrative Perspective on Incretin-Based Therapies

**Figure 5:**
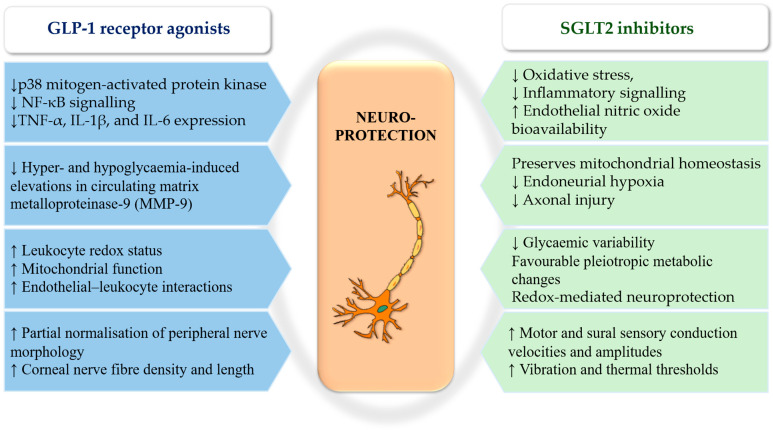
**Indirect neuroprotective mechanisms of GLP-1 receptor agonists and SGLT2 inhibitors in diabetic neuropathy.** This figure summarises the indirect neuroprotective effects of incretin-based therapies and sodium–glucose cotransporter-2 inhibitors in diabetic neuropathy. GLP-1 receptor agonists modulate inflammatory and stress-activated signalling pathways, including suppression of p38 mitogen-activated protein kinase and NF-κB activity, leading to reduced pro-inflammatory cytokine expression and improved redox and mitochondrial function. Structural and small-fibre endpoints, such as corneal nerve fibre parameters, show partial normalisation. SGLT2 inhibitors exert complementary effects through reduction in oxidative and inflammatory stress, improvement in endothelial nitric oxide bioavailability, and preservation of mitochondrial homeostasis, thereby attenuating endoneurial hypoxia and axonal injury. Together, these pleiotropic actions translate into improvements in electrophysiological and sensory outcomes, supporting a role for both drug classes as system-level, indirect neuroprotective strategies beyond glucose lowering alone. Abbreviations: GLP-1 RA, glucagon-like peptide-1 receptor agonist; SGLT2, sodium–glucose cotransporter-2; NF-κB, nuclear factor kappa B; TNF-α, tumour necrosis factor alpha; IL-1β, interleukin-1 beta; IL-6, interleukin-6; MMP-9, matrix metalloproteinase-9.

Collectively, DPP-4is and GLP-1 RAs represent the most promising pleiotropic candidates evaluated to date for diabetic neuropathy with emerging electrophysiological and structural signals suggesting possible neuroprotective effects, although these findings remain preliminary and require confirmation in neuropathy-specific randomised trials [94,95]. However, definitive conclusions regarding therapeutic efficacy await high-powered, neuropathy-focused, randomised, controlled trials using standardised endpoint groups, including pain assessment, small-fibre structure and function, nerve conduction measurements and autonomic indices [98] (Figure 5).

### 3.4. SGLT2 Inhibitors as Pleiotropic Neuroprotective Agents

Sensorimotor and autonomic diabetic neuropathy are driven by oxidative stress, inflammation, mitochondrial dysfunction and microvascular impairment. These conditions may be beneficially affected by SGLT2i. Beyond glucose lowering, SGLT2 inhibition attenuates oxidative stress, suppresses inflammatory signalling, improves endothelial nitric oxide bioavailability, and preserves mitochondrial homeostasis, mechanisms directly implicated in endoneurial hypoxia and axonal injury [101].

A three-year observational follow-up study reported that SGLT2i therapy improved composite neurophysiological scores, including motor and sural sensory conduction velocities and amplitudes, vibration and thermal thresholds, in patients with T2D. These improvements were associated with reduced glycaemic variability and favourable pleiotropic metabolic changes, suggesting mechanisms beyond glucose control [102]. In a randomised, placebo-controlled trial, empagliflozin improved electrophysiological parameters while reducing malondialdehyde levels without altering HbA1c, providing direct evidence for redox-mediated neuroprotection independent of glycaemic effects [103]. Similarly, a 20-week double-blind randomised trial in patients with established diabetic neuropathy demonstrated that add-on empagliflozin (10 mg/day) significantly improved Michigan Neuropathy Screening Instrument scores and peroneal nerve conduction velocity, alongside favourable renal functional changes [104].

Autonomic outcomes further support the pleiotropic potential of SGLT2 inhibition. Improvements in myocardial sympathetic innervation were also observed using ^123^I-metaiodobenzylguanidine scintigraphy, accompanied by reduced syncope recurrence at 12 months [105]. An observational study focusing on autonomic function showed that empagliflozin improved parasympathetic cardiac modulation, reflected by enhanced respiratory sinus arrhythmia amplitude and increased normal-to-normal interbeat intervals, and reduced the likelihood of QTc prolongation [106]. These effects suggest potential autonomic stabilisation, particularly relevant in patients with diabetic autonomic neuropathy receiving concomitant antiarrhythmic therapy.

In addition, an exploratory randomised open-label crossover trial in adults with T1D and established cardiovascular autonomic neuropathy demonstrated that short treatment periods with empagliflozin induced modest but measurable improvements in heart rate variability indices and cardiovascular autonomic reflex tests [107]. In a subsequent double-blind, randomised study in patients with T2D, canagliflozin was observed to augment the expression of antioxidant enzyme genes, including superoxide dismutase, catalase and glutathione peroxidase, in circulating CD34+ progenitor cells. This finding is consistent with an increase in glutathione-related defences and a reduction in oxidative stress [108]. Although preliminary, these data suggest that SGLT2i may favourably modulate autonomic balance across diabetes phenotypes, warranting targeted investigation in autonomic neuropathy-focused trials.

Experimental studies indicate that SGLT2 inhibition reduces mitochondrial ROS generation, enhances electron transport chain efficiency and promotes mitochondrial biogenesis through activation of the PGC-1α/mitochondrial transcription factor A axis [109,110]. A recent study in humans demonstrated improved endothelial function and reduced mitochondrial ROS production in endothelial cells following empagliflozin therapy, leading to increased NO bioavailability and improved microcirculatory perfusion [106]. A prospective translational study in patients initiating SGLT2i therapy demonstrated significant reductions in urinary ortho- and meta-tyrosine/para-tyrosine ratios and hydroxyl radical activity, accompanied by early improvements in sensory thresholds in individuals with small-fibre dysfunction. Correlations between redox marker changes and neuropathic measures provide direct human evidence for a redox-mediated neural benefit [111].

The safety profile of SGLT2i is well established in T2D. However, the risk of euglycemic diabetic ketoacidosis remains a limiting factor for T1D patients and necessitates cautious patient selection and monitoring. Importantly, neuropathy-focused cohorts in T2D have not demonstrated excess autonomic instability or clinically significant ketone dysregulation [112]. Safety considerations underscore the need for phenotype-specific application of SGLT2i, particularly in patients with advanced autonomic dysfunction or T1D.

Clinically, treatment with SGLT2 inhibitors in T2D has been associated with modest yet consistent improvements in neurophysiological conduction parameters and autonomic indices, accompanied by reductions in biomarkers of oxidative stress [102,103,105]. Beyond metabolic benefits, SGLT2 inhibition modulates mitochondrial function and redox homeostasis, including reductions in mitochondrial ROS generation and activation of mitochondrial biogenesis pathways. These effects are mediated, at least in part, through PGC-1α-dependent signalling and improved cellular energetic efficiency [109,113].

#### Positioning SGLT2 Inhibitors Within the Therapeutic Landscape

When combined with data from clinical studies on peripheral neuropathy and exploratory studies targeting cardiovascular autonomic neuropathy, this experimental and clinical evidence may support the hypothesis that SGLT2 inhibitors may influence neuropathy-relevant pathophysiological mechanisms, although definitive disease-modifying effects have not yet been demonstrated [102,103,104,107]. Nevertheless, definitive confirmation requires adequately powered, neuropathy-focused randomised controlled trials employing harmonised endpoint panels that integrate neuropathic pain assessment, small-fibre structure and function, nerve conduction studies and autonomic measures (Figure 5).

### 3.5. Targeting Upstream Metabolic Pathways

Chronic hyperglycaemia shifts glucose flux toward alternative metabolic pathways, resulting in enhanced oxidative stress, microvascular dysfunction and endoneurial injury. Two historically prominent therapeutic targets emerging from this framework are inhibition of the polyol pathway via aldose reductase inhibitors (ARIs) and modulation of PKC-β signalling. Both strategies are supported by mechanistic rationale but have encountered substantial translational and regulatory barriers.

#### 3.5.1. Aldose Reductase Inhibitors

Epalrestat, the most extensively studied ARI, attenuates sorbitol accumulation and reduces redox stress in peripheral nerves [114]. Beyond polyol pathway inhibition, epalrestat enhances endogenous antioxidant defences by increasing intracellular glutathione in Schwann cells through induction of Nrf2-dependent γ-glutamylcysteine ligase, while activating glutathione-, thioredoxin- and heme oxygenase-1-related programmes in endothelial cells [115,116]. These effects establish a direct mechanistic link between aldose reductase inhibition and restoration of redox homeostasis.

In clinical practice, epalrestat has been implemented in parts of Asia for the treatment of DSPN. In a 12-week, double-blind, randomised controlled trial, epalrestat improved spontaneous pain relief, nerve conduction velocity and sensory thresholds compared with placebo [114]. A three-year multicentre ADCT study further suggested delayed neuropathy progression with good tolerability, particularly among patients achieving improved glycaemic control [117]. A meta-analysis of 20 randomised controlled trials reported additive benefits of combination therapy, with ALA plus epalrestat outperforming either monotherapy across composite efficacy and conduction endpoints [118].

Despite the presence of plausible pathomechanistic rationale and a multitude of positive clinical observations, the application of ARIs to the standard treatment of DSPN remains limited. Early-generation agents (e.g., sorbinil, tolrestat, and zenarestat) were withdrawn from the market due to significant toxicity, undermining confidence in the drug class. A Cochrane review encompassing 32 trials found no statistically significant benefit over placebo on key neurological outcomes and identified major methodological and safety concerns [119]. Moreover, epalrestat remains licenced almost exclusively in Japan, but no contemporary, large-scale phase III trials with patient-centred endpoints have been completed. The development of newer ARIs, such as raniresstat and fidarestat, has largely stalled [120]. Although epalrestat demonstrates coherent redox-restorative mechanisms and reproducible symptomatic benefit, the absence of robust, modern phase III outcome data and limited global availability preclude classification of ARIs as disease-modifying therapies in diabetic neuropathy.

#### 3.5.2. PKC-β Inhibition

In contrast, clinical development of the PKC-β inhibitor ruboxistaurin has yielded inconsistent results. Hyperglycaemia-induced DAG accumulation activates PKC-β, contributing to oxidative stress, endothelial dysfunction and impaired endoneurial perfusion [121]. Ruboxistaurin, a selective PKC-β inhibitor, demonstrated improvements in microvascular blood flow, reductions in total neuropathy symptom scores and modest quality-of-life benefits in phase II trials spanning 6–12 months [122,123]. However, these effects were variable and largely confined to subgroups with less advanced neuropathy. A systematic review of six randomised controlled trials concluded that heterogeneity in study design, endpoint selection and small effect sizes precluded firm conclusions regarding clinical efficacy [124]. Importantly, no positive phase III development programme has emerged and regulatory approval has not been achieved.

#### 3.5.3. Integrative Perspective on ARIs and PKC Inhibitors

Both aldose reductase and PKC-β inhibition are supported by compelling mechanistic rationale and early clinical signals. However, limitations related to safety concerns, inconsistent efficacy, geographic availability, and lack of definitive phase III outcome trials have prevented their incorporation into international guideline-level therapy. Consequently, ARIs remain adjunctive options in selected clinical settings, while PKC-β inhibition persists as an experimental strategy pending evidence of a clinically meaningful, durable benefit [119,121,125].

## 4. Combination Strategies and Trial Design Considerations

The convergence of metabolic dysregulation, oxidative stress, inflammation, mitochondrial dysfunction, and microvascular impairment provides a strong biological rationale for combination strategies targeting complementary pathogenic mechanisms rather than isolated pathways. Consequently, such approaches have gained interest as pragmatic adjuncts to standard care in diabetic neuropathy.

### 4.1. Antioxidant–Neurotrophic Combinations

Adjunctive therapy combining ALA and methylcobalamin has been evaluated in short-term clinical studies. A meta-analysis reported that combined treatment for 2–4 weeks resulted in greater improvements in neuropathic symptoms and nerve conduction velocity compared with methylcobalamin monotherapy, without an increase in serious adverse events [126]. From a mechanistic standpoint, methylcobalamin promotes axonal regeneration and remyelination through neurotrophic signalling, whereas ALA primarily enhances mitochondrial redox balance and endothelial function. Human studies further indicate favourable shifts in ADMA, nitric oxide metabolism and inflammatory markers during ALA therapy, consistent with improved vascular and oxidative homeostasis [56,58]. In summary, the ALA–methylcobalamin combination represents a biologically coherent and clinically pragmatic adjunct, although evidence for sustained disease modification remains insufficient.

### 4.2. Polyol Pathway and Redox Modulation

Combination targeting of polyol pathway flux and oxidative stress has been explored using ALA and epalrestat. A meta-analysis of 20 randomised controlled trials demonstrated the superiority of this combination over either monotherapy in composite clinical efficacy and multiple nerve conduction outcomes, with acceptable tolerability [118]. These results support the dual targeting of polyol pathway flux reduction and oxidative stress regulation. Dual inhibition of sorbitol accumulation and oxidative injury provides mechanistic synergy; however, limitations in trial quality and the lack of contemporary long-term outcome data constrain definitive conclusions regarding disease modification.

### 4.3. Antioxidants Combined with Symptomatic Therapies

Combination approaches pairing antioxidants with established symptomatic agents have also yielded additive benefits. In a randomised, placebo-controlled trial in painful DSPN, addition of coenzyme Q10 to pregabalin resulted in greater pain relief, improved sleep disturbance scores and higher ≥50% responder rates after eight weeks [64]. Similarly, NAC administered alongside pregabalin reduced neuropathic pain and sleep disturbances in an eight-week double-blind trial, accompanied by reductions in malondialdehyde and increases in antioxidant defence markers, including superoxide dismutase, glutathione peroxidase, total antioxidant capacity, and thiol levels [66]. These combinations suggest an additive symptomatic benefit mediated by redox modulation but do not yet demonstrate structural or long-term disease-modifying effects.

### 4.4. Methodological Considerations for Future Combination Trials

The integration of patient-centred outcomes with objective neurophysiological measures has the potential for exploration in future combination therapy trials, which may yield insights into the multidimensional pathomechanism of diabetic neuropathy. Validated pain and sleep disturbance scales should be combined with corneal confocal microscopy to assess small-fibre pathology [127], alongside standardised nerve conduction studies for large-fibre dysfunction [3]. Importantly, trial designs should distinguish early small-fibre-predominant disease from advanced large-fibre neuropathy, as regenerative capacity and therapeutic responsiveness are likely stage-dependent. Incorporation of oxidative and redox biomarkers, including glutathione status, lipid peroxidation indices, antioxidant enzyme activity, and carbonyl stress markers, may facilitate mechanistic interpretation by linking biochemical changes to clinical responses [56,58,64,66].

### 4.5. Integrative Perspective on Combination Strategies

Taken together, combination therapies reflect a rational response to the multifactorial pathogenesis of diabetic neuropathy and demonstrate reproducible short-term symptomatic benefits. However, persistent heterogeneity in study design, short treatment durations and lack of definitive structural endpoints currently preclude classification of these strategies as disease-modifying. Accordingly, combination therapies should presently be regarded as adjuncts to standard diabetic neuropathy management, pending confirmation from adequately powered, mechanism-based randomised controlled trials employing harmonised clinical, neurophysiological, and biomarker endpoint [3,118] (Table 2).

## 5. Emerging and Investigational Approaches

Beyond established antioxidant and pleiotropic strategies, several additional or investigational approaches have been explored in diabetic neuropathy. These interventions vary widely in mechanistic specificity, clinical maturity, and translational relevance and should therefore be interpreted within a clearly defined evidentiary context.

### 5.1. Actovegin

Actovegin is a deproteinized hemoderivative derived from calf blood, containing low-molecular-weight peptides, amino acids, and metabolic intermediates. It is proposed to enhance cellular oxygen utilisation and energy metabolism. In a large, intravenous-to-oral, double-blind, randomised controlled trial in patients with T2D, actovegin improved neuropathic symptom scores, selected sensory domains, and quality-of-life measures [128]. However, the heterogeneity of outcome variables, absence of a clearly defined redox- or pathway-specific mechanism, and lack of structural or biomarker-based endpoints limit interpretation of disease-modifying potential. Actovegin may provide a symptomatic benefit in selected patients, but its uncertain mechanistic specificity precludes classification as a disease-modifying therapy in diabetic neuropathy.

### 5.2. Conventional Antioxidant Vitamins

Conventional antioxidant vitamins, including vitamins C and E, have been extensively evaluated in randomised clinical trials for diabetic neuropathy. Overall, results have been inconsistent or inconclusive, with no reproducible evidence of a sustained neurophysiological or structural benefit [3,129]. Consequently, contemporary reviews and international clinical guidelines do not recommend routine use of these agents for disease modification [70].

### 5.3. Nuclear Factor Erythroid 2-Related Factor 2 Activators

Activation of Nrf2 represents an attractive upstream strategy for inducing endogenous antioxidant defence programmes. Compounds such as sulforaphane have demonstrated robust activation of Nrf2 signalling and protection against oxidative injury in experimental models of diabetes [130]. However, randomised clinical trials focusing on neuropathy are lacking and current evidence remains confined largely to preclinical or early translational settings [10].

### 5.4. Poly(ADP-Ribose) Polymerase Inhibition

Inhibition of PARP targets the interface between oxidative DNA damage, NAD^+^ depletion and sterile inflammation and has demonstrated consistent neuroprotective effects in experimental models of diabetic neuropathy [131,132]. These findings position PARP as a mechanistically coherent upstream target linking redox stress to energetic failure. However, the human data in diabetic neuropathy remain limited and future clinical trials should include pharmacodynamic measures that directly link PARP modulation to both experimental and clinical outcomes.

### 5.5. Integrative Perspective on Investigational Approaches

Collectively, these investigational and contextual strategies underscore the persistent gap between mechanistic plausibility and clinical validation in diabetic neuropathy. While several approaches demonstrate biological coherence and preclinical efficacy, the lack of neuropathy-specific, well-powered clinical trials with harmonised endpoints remains the principal barrier to translation. Accordingly, these interventions should currently be regarded as exploratory or adjunctive, while future research prioritises mechanism-driven trial designs capable of distinguishing symptomatic modulation from true disease modification.

## 6. Discussion

Diabetic neuropathy emerges from the convergence of disturbances in redox balance, mitochondrial function, inflammatory signalling, and microvascular regulation [7,28]. This integrative pathophysiological framework provides a coherent rationale for therapeutic strategies that address interconnected mechanisms rather than isolated downstream targets. Within this context, classical antioxidants such as α-lipoic acid and acetyl-L-carnitine have demonstrated reproducible symptomatic benefits and modest neurophysiological improvements [47,62] and may influence early pathogenic processes when applied at appropriate stages of disease [54,58]. However, their long-term disease-modifying efficacy remains unconfirmed.

Targeted metabolic interventions, including aldose reductase inhibitors and PKC-β inhibitors, further address hyperglycaemia-driven oxidative and vascular injury. Early clinical signals, particularly with epalrestat, suggest symptomatic and electrophysiological benefits [117,123]. Nevertheless, historical safety concerns, limited geographic availability and the absence of contemporary confirmatory phase III trials have constrained their incorporation into international treatment paradigms [119,124].

**Figure 6:**
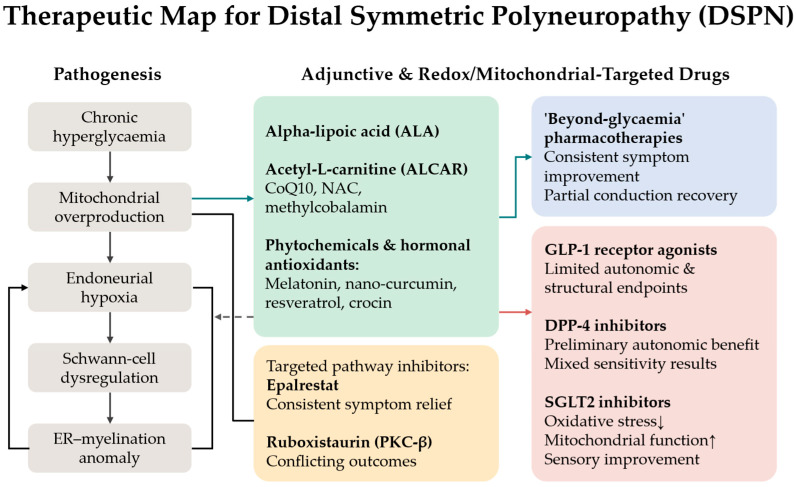
**Integrated pathogenetic–therapeutic framework for distal symmetric polyneuropathy (DSPN).** This figure presents an integrated pathogenetic and therapeutic map of DSPN, highlighting mitochondrial dysfunction and oxidative stress as central downstream consequences of chronic hyperglycaemia. These processes contribute to endoneurial hypoxia, Schwann-cell dysregulation, and endoplasmic reticulum-related myelination abnormalities, collectively driving axonal damage. Adjunctive redox- and mitochondria-targeted therapies primarily aim to attenuate oxidative stress and support mitochondrial function, resulting in relatively consistent symptomatic improvement but variable structural recovery. Targeted pathway inhibitors address specific hyperglycaemia-activated mechanisms with heterogeneous clinical outcomes. In parallel, so-called “beyond-glycaemia” pharmacotherapies exert indirect neuroprotective effects through systemic metabolic optimisation and pleiotropic actions, including improvements in oxidative stress, mitochondrial efficiency, and sensory function. Overall, the framework emphasises the need for mechanism-aligned, stage-specific combination strategies rather than single-pathway interventions. Abbreviations: DSPN, distal symmetric polyneuropathy; ER, endoplasmic reticulum; ALA, α-lipoic acid; ALCAR, acetyl-L-carnitine; CoQ10, coenzyme Q10; NAC, N-acetylcysteine; PKC-β, protein kinase C beta; GLP-1, glucagon-like peptide-1; DPP-4, dipeptidyl peptidase-4; SGLT2, sodium–glucose cotransporter-2.

In contrast, pleiotropic antidiabetic agents such as SGLT2is [109,111] and GLP-1 RAs [90,94] exert broad effects on mitochondrial function, oxidative stress, inflammatory signalling and endothelial integrity. Emerging human data indicate modest yet consistent improvements across neuropathic symptoms, neurophysiological measures and autonomic indices, with effects that appear at least partially independent of glycaemic control [105]. Collectively, these findings position such agents as biologically plausible candidates capable of influencing multiple pathogenic pathways involved in diabetic neuropathy, although neuropathy-specific clinical evidence remains limited (Figure 6).

Methodological advances now allow for substantially more precise evaluation of therapeutic effects through multidimensional endpoint frameworks paired with validated biochemical and structural biomarkers. Comprehensive phenotyping, integrating patient-reported pain and sleep outcomes, quantitative sensory testing, nerve conduction studies and small-fibre imaging using corneal confocal microscopy [9,127], enables detection of early neural injury and monitoring of treatment response across both distal symmetric and autonomic neuropathy. Integrating oxidative and inflammatory markers, including lipid peroxidation indices and carbonyl stress markers, further strengthens causal inference by linking mechanistic modulation to clinical outcomes [133,134].

The rationale for the clinical administration of actovegin, Nrf2 activators, and PARP inhibitors is supported by biologically coherent mechanisms, including the targeting of redox imbalance, mitochondrial dysfunction, and NAD^+^ depletion. However, the current clinical validation of these mechanisms remains limited [128,130,131,132]. The majority of available data are derived from preclinical studies or exploratory translational evidence, and there is a significant paucity of adequately powered, neuropathy-specific, randomised, controlled trials with prespecified structural and functional outcomes [10]. It is imperative to note that enhancements in surrogate biomarkers or short-term symptomatic measures may not be interpreted as substantiated evidence of sustained neuroregeneration or confirmed disease modification, particularly in the absence of consistent small-fibre structure assessment (e.g., corneal confocal microscopy) and harmonised endpoint panels [9,127]. Consequently, despite the fact that these strategies continue to demonstrate significant mechanistic potential, their definitive therapeutic positioning necessitates the execution of rigorously designed, phased clinical trials encompassing multidimensional neuropathy endpoints [10].

Together with growing recognition of the importance of early metabolic and vascular intervention, these methodological developments create favourable conditions for evaluating coordinated combination strategies that simultaneously target metabolic, redox, and neurovascular pathways [125]. Ultimately, harmonising mechanistic insight with neuropathy-specific clinical trial frameworks represents a credible pathway toward interventions capable of altering the long-term trajectory of diabetic neuropathy, rather than merely alleviating symptoms [48].

Future clinical investigation in diabetic neuropathy should shift from predominantly symptom-oriented designs toward mechanism-aligned, neuropathy-specific trial frameworks with harmonised endpoint panels. We propose a multidimensional approach to endpoint selection, integrating patient-centred outcomes (pain and quality of life), large-fibre electrophysiology (standardised nerve conduction parameters), small-fibre structure and function (e.g., corneal confocal microscopy and other small-fibre measures), and autonomic testing where cardiovascular autonomic neuropathy is targeted [3,70,105,106,107,127]. The mechanistic interpretation should be strengthened by incorporating redox- and oxidative stress-related biomarker panels that can be linked to functional outcomes. This is in accordance with human translational studies that demonstrate correlations between shifts in redox markers and neuropathic measures [56,58,64,66,111].

Given the convergence of oxidative stress, mitochondrial dysfunction, inflammation, and microvascular impairment, it is recommended that rational combination strategies be tested within hypothesis-driven randomised designs rather than empiric polypharmacy. Clinically explored combinations (e.g., antioxidant–neurotrophic or polyol pathway–redox modulation, as well as antioxidant add-on to standard analgesic regimens) provide a mechanistic rationale for structured evaluation [64,66,118,126]. It is crucial to implement stage-specific stratification to avoid the dilution of treatment signals, with the objective of distinguishing early small-fibre-predominant neuropathy from mixed phenotypes and advanced large-fibre neurodegeneration. This is because regenerative capacity and therapeutic responsiveness are stage-dependent [3,127]. Ultimately, the duration of the trial should be adequate to observe structural change, as short-term symptomatic improvement is unlikely to reflect enduring disease modification. Longer-term studies remain essential to define clinically meaningful progression slowing or regeneration [49,60,117].

This review has several limitations that merit consideration. First, as a narrative rather than a systematic review, it does not aim to exhaustively capture all available evidence and selection bias in the included literature cannot fully be excluded. Second, the clinical studies discussed are characterised by substantial heterogeneity in design, sample size, neuropathy phenotype, treatment duration, and outcome measures, limiting cross-study comparability and precluding definitive quantitative synthesis. Third, multiple antioxidant and pathway-specific strategies demonstrate strong mechanistic plausibility, but the majority lack adequately powered, neuropathy-specific randomised controlled trials with standardised structural and functional endpoints. Moreover, short study duration, heterogeneous endpoint selection, and insufficient stage stratification currently limit the ability to distinguish symptomatic benefits from true disease modification. Forth, although mechanistic data strongly support targeting oxidative stress and related pathways, translation into confirmed disease-modifying clinical efficacy remains limited by the scarcity of large, neuropathy-focused randomised controlled trials with harmonised endpoints. Finally, several promising therapeutic avenues, including novel antioxidant compounds, combination regimens, and emerging epigenetic or metabolic modulators, remain in early preclinical or exploratory phases and require rigorous validation before consideration for routine clinical application.

## 7. Conclusions

The convergence of oxidative stress, chronic inflammation, mitochondrial dysfunction and microvascular impairment highlights diabetic neuropathy as a multifactorial condition driven by interacting pathogenic mechanisms rather than a single dominant pathway. Oxidative stress plays a key role in linking metabolic disturbances with vascular and neuronal injury, supporting the rationale for therapeutic approaches that extend beyond isolated antioxidant supplementation. Classical antioxidants and mitochondrial-supportive agents, including α-lipoic acid, acetyl-L-carnitine, and coenzyme Q10, have shown reproducible symptomatic benefits, particularly in neuropathic pain; however, robust evidence for sustained disease-modifying effects remains limited, especially in advanced stages of neuropathy.

In contrast, pleiotropic antidiabetic therapies, including SGLT2is and GLP-1 RAs, exert multiple effects on oxidative stress, inflammation, endothelial function and mitochondrial processes. Available clinical data indicate modest but consistent improvements in selected neurophysiological and autonomic parameters, which appear to be at least partly independent of glycaemic control. Further progress in this field will require neuropathy-specific, adequately powered randomised controlled trials incorporating standardised clinical endpoints and validated mechanistic biomarkers to determine whether these agents may modify the long-term course of diabetic neuropathy beyond symptomatic benefits.

## Figures and Tables

**Table 2:** Therapeutic strategies targeting oxidative stress in diabetic neuropathy: mechanistic rationale and clinical evidence.

Therapeutic Class	Representative Agents	Primary Antioxidant/ Pleiotropic Mechanisms	Clinical Effects in Diabetic Neuropathy	Limitations and Interpretative Considerations	References
Classical antioxidants	α-Lipoic acid	Direct ROS scavenging; regeneration of endogenous antioxidants; improved endoneurial perfusion	Reproducible reduction in neuropathic pain; modest improvements in oxidative biomarkers	Inconsistent effects on nerve conduction and structural endpoints; limited evidence for durable disease modification	[46,47,48,49,50,51,52,56,57,58,59,60]
Mitochondrial-supportive agents	Acetyl-L-carnitine	Enhancement of mitochondrial β-oxidation; neurotrophic signalling; improved axonal energetics	Modest analgesic benefit; small improvements in nerve conduction velocity	Heterogeneous trial designs; low certainty of long-term efficacy	[61,62,63]
Electron transport cofactors	Coenzyme Q10	Improved electron transport chain efficiency; reduction in mitochondrial ROS	Additive analgesic effects when combined with standard symptomatic therapy	Limited stand-alone data; structural benefit unproven	[64,65]
Glutathione-augmenting agents	N-Acetylcysteine	Restoration of intracellular glutathione; suppression of oxidative and inflammatory signalling	Reduced neuropathic pain and oxidative stress markers in short-term trials	Evidence confined to small, short-duration studies	[66,67]
Vitamin-based neurotrophic support	Methylcobalamin (vitamin B12)	Promotion of axonal regeneration and remyelination	Improvement in neuropathic symptoms and selected neurophysiological parameters	Benefits most evident in deficiency states or combination regimens	[8,68,69]
Phytochemicals	Curcumin, resveratrol, crocin	Activation of endogenous antioxidant pathways (e.g., Nrf2); inhibition of NF-κB signalling	Symptomatic improvement in selected trials; inconsistent neurophysiological effects	Variable bioavailability; inconsistent clinical outcomes	[76,77,78,79,80,81,82,83,84,85]
Hormonal antioxidants	Melatonin	Free radical scavenging; modulation of nociceptive and circadian pathways	Improvement in pain and sleep disturbance as adjunctive therapy	Disease-modifying effects not demonstrated	[71,72,73,74,75]
Incretin-based therapies	GLP-1 receptor agonists; DPP-4 inhibitors	Attenuation of oxidative stress and inflammation; improved mitochondrial and endothelial function	Emerging improvements in nerve conduction and autonomic indices	Limited neuropathy-focused randomised trials; phenotype-specific effects	[87,88,89,90,91,92,93,94,95,98]
SGLT2 inhibitors	Empagliflozin; canagliflozin	Reduction in mitochondrial ROS; improved endothelial NO bioavailability; enhanced mitochondrial biogenesis	Consistent improvements in neurophysiological and autonomic measures independent of glycaemic control	Confirmation required in adequately powered, neuropathy-specific trials	[101,102,103,104,105,106,107,108,109,110,111,113]
Upstream metabolic pathway inhibitors	Aldose reductase inhibitors; PKC-β inhibitors	Reduction in polyol- and PKC-mediated oxidative stress	Symptomatic and electrophysiological benefits in early trials	Safety concerns; inconsistent efficacy; lack of contemporary phase III data	[114,115,116,117,118,119,120,121,122,123,124]

This table summarises therapeutic strategies targeting oxidative stress in diabetic neuropathy across different levels of the pathogenic cascade. Classical antioxidants and mitochondrial-supportive agents predominantly provide symptomatic relief and short-term improvements in oxidative stress markers, with limited evidence for sustained disease modification. In contrast, pleiotropic antidiabetic therapies, particularly incretin-based agents and sodium–glucose cotransporter-2 inhibitors, modulate oxidative stress, inflammation, mitochondrial function, and endothelial integrity in parallel, thereby offering broader mechanistic coverage and emerging potential for structural and functional benefits. Abbreviations: DPP-4, dipeptidyl peptidase-4; GLP-1, glucagon-like peptide-1; NF-κB, nuclear factor kappa B; NO, nitric oxide; Nrf2, nuclear factor erythroid 2-related factor 2; PKC-β, protein kinase C beta isoform; ROS, reactive oxygen species; SGLT2, sodium–glucose cotransporter-2.

## Data Availability

No new data were created or analyzed in this study. Data sharing is not applicable to this article.

## Supplementary Materials

The following supporting information can be downloaded at: https://www.mdpi.com/article/10.3390/antiox15030367/s1.

## Abbreviations

The following abbreviations are used in this manuscript:

ADMA	Asymmetric dimethylarginine
AGE	Advanced glycation end product
ALA	Alpha-lipoic acid
ALCAR	Acetyl-L-carnitine
ARI	Aldose reductase inhibitor
CHOP	C/EBP homologous protein
DAG	Diacylglycerol
DPP-4i	Dipeptidyl peptidase-4 inhibitor
DSPN	Distal symmetric polyneuropathy
eNOS	Endothelial nitric oxide synthase
ER	Endoplasmic reticulum
GFAT	Fructose-6-phosphate amidotransferase
GLP-1 RA	Glucagon-like peptide-1 receptor agonist
MMP-9	Matrix metalloproteinase-9
NAC	N-acetylcysteine
NAD^+^	Nicotinamide adenine dinucleotide
NADPH	Nicotinamide adenine dinucleotide phosphate
Nav	Voltage-gated sodium channels
NF-κB	Nuclear factor kappa B
NLRP3	NOD-like receptor protein 3
Nrf2	Nuclear factor erythroid 2-related factor 2
PAI-1	Plasminogen activator inhibitor-1
PARP	Poly(ADP-ribose) polymerase
PERK	Protein kinase RNA-like ER kinase
PGC-1α	Peroxisome proliferator-activated receptor gamma coactivator-1 alpha
PKC	Protein kinase C
RAGE	Receptor for advanced glycation end products
ROS	Reactive oxygen species
SGLT2i	Sodium–glucose cotransporter-2 inhibitor
T1D	Type 1 diabetes mellitus
T2D	Type 2 diabetes mellitus
TGF-β	Transforming growth factor beta
TNF-α	Tumour necrosis factor alpha
UPR	Unfolded protein response
VEGF	Vascular endothelial growth factor
