# Supplementary material for: Targeting Oxidative Stress and Mitochondrial Dysfunction in Diabetic Neuropathy: Mechanisms and Therapeutic Opportunities
Source: Antioxidants (Basel). 2026 Mar 13;15(3):367. doi: 10.3390/antiox15030367 (PMC13024170; doi:10.3390/antiox15030367)
Supplement: Supplementary file 1 [file antioxidants-15-00367-s001.zip › antioxidants-4175281-supplementary.pdf]

Supplementary Text S1.

Methodological Workflow for Literature Review and Evidence Grading

Overview

This supplementary text documents the full workflow used for literature identification, data extraction, evidence grading, and figure/table integration in the review *"Targeting Oxidative Stress and Mitochondrial Dysfunction in Diabetic Neuropathy: Mechanisms and Therapeutic Opportunities"*.
The approach follows **PRISMA-2020** recommendations for narrative reviews.

1. Search Protocol

Databases searched:
PubMed (MEDLINE), Scopus, and Web of Science.

Timeframe:
January 2010 – June 2025 (updated June 2025).

Search string:

("diabetic neuropathy" OR "distal symmetric polyneuropathy" OR "DPN" OR "DSPN")
AND ("oxidative stress" OR "mitochondrial dysfunction" OR "redox imbalance")
AND ("antioxidant therapy" OR "α-lipoic acid" OR "acetyl-L-carnitine" OR "coenzyme Q10" OR "N-acetylcysteine" OR "vitamin B12" OR "melatonin" OR "curcumin" OR "resveratrol" OR "crocin" OR "GLP-1" OR "SGLT2" OR "aldose reductase" OR "PKC")

Filters applied:

- English language
- Human or validated diabetic animal model
- Full-text availability

Manual search:
Reference lists of major meta-analyses, guidelines (Pop-Busui et al., 2017; Feldman et al., 2019), and recent mechanistic reviews (Savelieff et al., 2020; Zhu 2024) were hand-screened.

2. Eligibility Criteria

Inclusion:

1. Experimental, translational, or clinical studies assessing oxidative/mitochondrial mechanisms or antioxidant/intervention efficacy in diabetic neuropathy.
2. Quantitative outcome measures (redox markers, neurophysiology, pain, or morphological indices).

Exclusion:

- Non-diabetic neuropathies
- Reviews without primary data
- Conference abstracts or non-peer-reviewed reports

3. Screening and Data Extraction

- **Stage 1:** Title/abstract screening by two independent reviewers.
- **Stage 2:** Full-text assessment for eligibility.
- **Stage 3:** Data extraction into structured Excel tables (study design, population, intervention, dose, duration, mechanistic/clinical endpoints).
- **Stage 4:** Verification of quantitative consistency and cross-reference to original figures/tables.
  Discrepancies were resolved through consensus.

4. Synthesis and Figure/Table Development

Data from included studies were stratified into three thematic layers:

1. **Pathophysiological mechanisms** (oxidative, mitochondrial, inflammatory, vascular nodes) – forming *Table 1* and *Figure 1*.
2. **Adjunctive redox–mitochondrial therapeutic strategies** (ALA, ALCAR, CoQ10, NAC, vitamins) – forming *Table 2* and *Figure 2*.
3. **Emerging agents** (incretins, SGLT2 inhibitors, ARIs/PKC inhibitors) – described narratively in *Section 5*.

6. Data Availability and Reproducibility

All extracted datasets, intermediate spreadsheets, and figure source files are available upon reasonable request. The complete workflow was stored in Zotero v6.0 and validated against PubMed IDs to ensure reference traceability.

PRISMA Flow Diagram of Literature Identification and Screening

Identification

- Records identified through database searching (PubMed = 624; Scopus = 411; Web of Science = 286) → **Total = 1321**
- Additional records identified through manual reference screening (meta-analyses + guidelines) → **n = 37**
  Total records before duplicates removed = 1358
- Duplicates removed → **n = 412**
  Records after deduplication = 946

Screening

- Titles/abstracts screened → **n = 946**
  • Excluded for irrelevance (non-diabetic neuropathy, unrelated antioxidant topic) = 703
  Full-text articles assessed for eligibility = 243

Eligibility

- Excluded full texts = 179
  • Review or commentary without primary data = 81
  • Conference abstract / non-peer-reviewed = 42
  • Lacking oxidative-stress or mitochondrial endpoints = 31
  • Non-English / unavailable full text = 25
  Studies included in qualitative synthesis = 64

Included

- Mechanistic / translational studies = 25
- Clinical randomized or controlled trials = 23
- Meta-analyses / pooled re-analyses = 8
- Observational human studies = 8
  Total included in final Systematic Narrative Review = 64

Search Record (PubMed + Scopus, January 2010 – June 2025)

**Database**	**Search Date**	**Search Query (String)**	**Filters Applied**	**Records Retrieved**	**Notes / Export Format**
**PubMed (MEDLINE)**	15 June 2025	("diabetic neuropathy"[MeSH Terms] OR "distal symmetric polyneuropathy" OR "DSPN" OR "DPN") AND ("oxidative stress" OR "mitochondrial dysfunction" OR "redox imbalance") AND ("antioxidant therapy" OR "α-lipoic acid" OR "acetyl-L-carnitine" OR "coenzyme Q10" OR "N-acetylcysteine" OR "vitamin B12" OR "melatonin" OR "curcumin" OR "resveratrol" OR "crocin" OR "GLP-1" OR "SGLT2"	English [lang]; Humans OR validated diabetic animal models; Full text available	**624**	Exported as .csv via PubMed "Send to → File → CSV"

Database	Search Date	Search Query (String)	Filters Applied	Records Retrieved	Notes / Export Format
		OR "aldose reductase" OR "PKC")			
**Scopus**	16 June 2025	TITLE-ABS-KEY(("diabetic neuropathy" OR "distal symmetric polyneuropathy" OR "DSPN") AND ("oxidative stress" OR "mitochondrial dysfunction") AND ("antioxidant" OR "α-lipoic acid" OR "coenzyme Q10" OR "GLP-1" OR "SGLT2"))	Article type: research article; Language: English; Year: 2010–2025	**411**	Results exported to Excel (.csv) with DOI and abstract fields
**Web of Science**	17 June 2025	TS=("diabetic neuropathy" AND "oxidative stress" AND ("antioxidant" OR "mitochondrial"))	Article; English; 2010–2025	**286**	Exported as BibTeX for Zotero import
**Manual Reference Search**	18 June 2025	Reference lists of Feldman 2019, Savelieff 2020, Ziegler 2021 (meta-analyses and guidelines)	None	**37**	Manual entry to Zotero library
**Total before deduplication**				**1358**	
**Duplicates removed**				**412**	Zotero “Find Duplicates” tool
**Records after deduplication**				**946**	Screening proceeded in Rayyan 2025 app

Documentation note:
All search exports (PubMed CSV, Scopus Excel, WoS BibTeX) and the final deduplicated Zotero library (in RIS format) are archived under the internal protocol **DSPN-OXSTRESS-**

2025-01 (University of Debrecen Systematic Review Registry) and are available upon request.
